# ASSISTED LIVING ADMINISTRATORS’ JOB SATISFACTION, WORK STRESSORS, AND INTENT TO LEAVE DURING THE COVID-19 PANDEMIC

**DOI:** 10.1093/geroni/igac059.2830

**Published:** 2022-12-20

**Authors:** Mary McSweeney-Feld, Teta Barry, Bo Yang, H Wayne Nelson

**Affiliations:** Towson University, Towson, Maryland, United States; Arcadia University, Glenside, Pennsylvania, United States; Towson University, Towson, Maryland, United States; Towson University, Towson, Maryland, United States

## Abstract

This study examines how job satisfaction in six sub-scales and selected stressors and demographic covariates influenced assisted living administrators’ (ALF) intentions to quit during the COVID-19 pandemic. Quantitative and qualitative data were collected from 103 ALF administrators as part of a national study of long-term care administrators’ intent to quit during the COVID-19 pandemic funded by the Foundation of the National Association of Long-Term Care Administrator Boards in Washington, DC. Descriptive statistics were collected for the sample, and correlations between variables were examined, as well as responses from 3 open-ended questions that were coded for analysis. Although generally satisfied, roughly 41 percent of ALF administrators reported that they were intending to quit. Qualitative data suggested that job satisfaction was influenced by a more nuanced interpretation of job characteristics and work environment intrinsic factors such as adequacy of staffing and resources, changing regulations during the COVID-19 pandemic, and external supports such as family and friends. Given the limited research on the impact of the COVID-19 pandemic on assisted living communities and their administrators, the results of this study can help to inform policies and strategies for providing support for this segment of long-term services and its workforce during widespread disasters.

